# Triple semicircular canal occlusion with endolymphatic sac decompression for intractable Meniere’s disease

**DOI:** 10.3389/fneur.2024.1362603

**Published:** 2024-04-17

**Authors:** Jiawang Tian, Gendi Yin, Qian Zhang, Shuqi Zhang, Xiangli Zeng, Yongqi Li

**Affiliations:** ^1^Department of Otorhinolaryngology-Head and Neck Surgery, The Third Affiliated Hospital of Sun Yat-Sen University, Guangzhou, China; ^2^Department of Otorhinolaryngology-Head and Neck Surgery, Guangzhou First People’s Hospital, Guangzhou, China

**Keywords:** Meniere’s disease, semicircular canal plugging, vertigo, hearing, endolymphatic sac decompression

## Abstract

**Background:**

Meniere’s disease (MD) is characterized by idiopathic endolymphatic hydrops (ELH). Frequent vertigo attacks is the most disabling symptom of MD.

**Objective:**

This study evaluated the efficacy of triple semicircular canal occlusion combined with endolymphatic sac decompression in the treatment of frequent vertigo in patients with MD.

**Methods:**

Eleven patients with complete medical records were included in this study conducted from May 2021 to April 2022. All patients were enrolled to undergo triple semicircular canal occlusion (TSCO) with endolymphatic sac decompression (ESD). Various tests including pure tone audiometry (PTA), vestibular evoked myogenic potentials (VEMPs), the video head impulse test (v-HIT), caloric test data, the Dizziness Handicap Inventory (DHI), the Berg Balance Scale (BBS), and the Tinnitus Handicap Inventory (THI) were performed both before and after the surgery.

**Results:**

The successful control rate of vertigo was 100% (9/9) in the average 23-month postoperative follow-up period, with complete control rate of 88.89% (8/9) and substantial control rate of 11.11% (1/9).

**Conclusion:**

Triple semicircular canal occlusion combined with ESD may be an effective treatment option for managing frequent vertigo attacks in patients with MD. This combination therapy has the potential to become a significant addition to the treatment framework for MD.

## Introduction

Meniere’s disease (MD) is a common inner ear disease characterized by idiopathic endolymphatic hydrops (ELH) (1) that results in attacks of spontaneous vertigo. MD is often accompanied by progressive sensorineural hearing loss, tinnitus, and aural fullness.

The current treatment strategies for MD begin with lifestyle interventions, followed by medical treatment, then the intratympanic injection of steroids (2). Although most MD patients have good vertigo control after >6 months of medical treatment, some patients have poor vertigo control. Frequent vertigo attacks is the most disabling symptom of MD, and often has a major negative impact on daily life and work. For patients who continue to experience disabling attacks, incapacitation, and unilateral involvement after 3–6 months of conservative management, treatment options include intratympanic gentamicin (ITG), intratympanic steroid (ITS), endolymphatic sac decompression (ESD), triple semicircular canal occlusion (TSCO), and vestibular nerve section (3, 4).

Semicircular canal occlusion, first described by Parnes and McClure (5), is a successful and safe surgical technique for benign paroxysmal positional vertigo (BPPV). Yin et al. (6) first advocated TSCO as a surgical treatment for MD. TSCO has since been shown to be an effective treatment strategy for MD. Based on the continued treatment of MD patients with TSCO by otologists, > 90% of patients have reported excellent postoperative outcomes of vertigo control (6–11). One study, however, reported that obstruction of the semicircular canal as a treatment for BPPV may result in endolymphatic hydrops (12).

The purpose of ESD is to decrease the high pressure in the endolymphatic sac. ESD is the classic surgical treatment for MD worldwide (13). Whether TSCO combined with ESD achieves good vertigo control and fewer side effects for intractable MD has not been established. Indeed, there are few reports on this issue worldwide.

The purpose of this study was to evaluate the efficacy of vertigo control using TSCO combined with ESD performed by our team of otology surgeons. Pre- and post-operative auditory function was measured to assess hearing preservation.

## Materials and methods

### Patient selection

The inclusion criteria were as follows: (1) previous standardized conservative treatment for at least 6 months without effective control of vertiginous symptoms, (2) no pathologic signs in the contralateral ear, and (3) a strong desire to undergo surgery in the hope of symptom resolution. The exclusion criteria were as follows: (1) possible bilateral MD; (2) middle ear disease; and (3) vestibular migraines, cerebellopontine angle tumors, transient ischemic attack, vestibular paroxysmia, recurrent unilateral vestibulopathy, and other vestibular disorders or any other disease that can cause vertigo.

A total of nine patients underwent surgical treatment. The patients presented with a primary complaint of frequent rotational vertigo attacks that was preceded by severe unilateral deafness. Prior to undergoing surgery, all patients underwent standardized conservative treatment for a minimum duration of 6 months, which included lifestyle modifications and medical interventions. The treatment regimen involved promoting healthy sleep patterns, reducing stress levels, abstaining from caffeine, alcohol, and tobacco, and adhering to a low-salt diet. Additionally, all patients were prescribed betahistine (12 mg three times a day) and hydrochlorothiazide (25 mg twice a day). The median age at the time of surgery was 56 years (age range, 49–67 years). All nine patients completed the follow-up evaluations after surgery.

### Auditory and vestibular function tests

Pure tone audiometry (PTA), vestibular evoked myopotentials (VEMPs), the video head impulse test (v-HIT), and caloric test data were collected before and after surgery to assess audiologic and vestibular function. Moreover, to evaluate the individual proportional decline and restoration, vestibular data were normalized to the preoperative value. VEMP abnormalities were defined as follows: cervical vestibular evoked myogenic potential (cVEMP), a p1 latency >17.3 ms, an n1 latency >24.6 ms, or an amplitude ratio > 30%; and ocular vestibular evoked myogenic potential (oVEMP), an n1 latency >12.6 ms, a p1 latency >17.8 ms, or an amplitude ratio > 30%. Other subjective evaluation information was completed by patients before and after surgery, including the dizziness handicap inventory (DHI), Berg Balance Scale (BBS), and the Tinnitus Handicap Inventory (THI).

### Evaluation of vertigo

According to the 1995 American Academy of Otolaryngology-Head and Neck Surgery guidelines (14), the vertigo control status was determined by calculating the frequency of vertigo attack episodes from 6 months before surgery to 18–24 months after surgery. Vertigo control ratio was calculated numerically by dividing the number of attacks after treatment by the number of attacks prior to treatment, then multiplied by 100. The following scale was used to express vertigo control: 0 = A, indicating complete control; 1–40 = B, indicating substantial control; 41–80 = C, indicating limited control; 81–120 = D, indicating insignificant control; and > 120 = E, indicating no control. The frequency of vertigo attacks 6 months after treatment was counted for patients who had undergone surgery <2 years earlier.

### Evaluation of hearing

Hearing function was assessed using the pure tone average (PTA), which measures the average hearing value at 0.5–1–2–4 kHz. A significant change was defined as a 10 dB difference between pre- and post-intervention measurements (14). A change in hearing level was defined as follows: worse, elevation ≥10 dB; better, decline ≥10 dB; and change within 0–10 dB, “no change.”

### Surgical technique

The main steps of the surgical procedure began performing a mastoidectomy with an intact posterior wall of the auditory canal through a postauricular incision under general anesthesia. Some temporalis fascia was harvested for later use. The horizontal semicircular canal, the posterior semicircular canal, and the superior semicircular were gradually skeletonized. The endolymphatic sac was identified inferior-to-posterior to the posterior semicircular canal, at the posterior fossa dura anterior-to-inferior to the sigmoid sinus, and below the extension line of the horizontal semicircular canal. The bone over the sac was widely removed to reveal the endolymphatic sac. The anterior wall of the endolymphatic sac was incised to provide sufficient decompression, and then steroids were instilled into the endolymphatic sac by immersion for 5 min.

Triple semicircular canal occlusion was then performed by skeletonizing the cavity, after which the three semicircular canals were contoured and gradually identified. Each canal was drilled up to the “blue line” and a fenestra, approximately 1 mm in diameter, was created. The fenestra was pried off using a hook, and then plugged with a piece of temporalis fascia. Subsequently, some bone dust was overlayed on the temporalis fascia, and then a larger piece of temporalis fascia was used to cover the bone dust. A gelatin sponge was then used to fill the surgical site. Similarly, the other canals were occluded in turn.

### Vestibular rehabilitation therapy

3–5 days after surgery, a vestibular rehabilitation program was initiated, including gaze stabilization exercises (GSEs), and balance and gait training. All patients were required to continue vestibular rehabilitation for at least 3 months postoperatively.

### Statistical analysis

The results were analyzed using the Student *t*-test. *p* values <0.05 were considered significant. All statistical analyses were performed using GraphPad Prism 8 software.

## Results

All patients had temporary vertigo within 3 days following the TSCO procedure combined with ESD. Moreover, all patients displayed spontaneous nystagmus immediately after the surgery and some patients complained of minor imbalance for several weeks. None of the patients had severe complications, such as facial paralysis, cerebrospinal fluid leakage, or infections. The patient characteristics, hearing, tinnitus function data, and vertigo function data are presented in Table 1.

**Table 1:** Pre- and post-operative clinical profiles.

Case no.	Age (years)	Side	Gender	Disease duration (years)	Hearing (PTA dB HL)			DHI			BBS			THI			Follow-up (months)	HYN	Smoking	TC (mmol/L)	FBG (mmol/L)
					Pre	1 M	18 M	Pre	1 M	18 M	Pre	1 M	18 M	Pre	1 M	18 M					
1	51	L	F	3	70	63	72	70	36	8	34	38	52	54	42	20	28	N	N	4.60	4.88
2	67	L	M	8	76	76	78	66	42	0	25	30	44	45	36	26	28	N	N	4.34	4.55
3	55	L	F	6	61	93	95	88	16	2	30	36	49	88	68	42	28	N	N	5.14	5.35
4	49	L	F	4	98	90	93	74	32	10	28	32	50	62	38	18	27	N	N	4.63	4.75
5	52	L	M	7	70	62	64	62	22	6	32	41	52	NT	NT	NT	26	N	N	5.81	5.46
6	63	L	F	4	66	81	76	90	34	20	28	34	38	46	38	20	20	N	N	5.40	4.87
7	61	L	F	7	62	86	70	94	66	26	26	32	43	58	46	32	20	Y	N	6.07	7.26
8	56	L	F	3.5	60	45	52	68	26	0	31	36	47	46	34	18	19	N	N	5.16	4.36
9	59	R	F	5	78	80	82	64	20	6	32	40	53	30	26	16	18	N	N	5.39	5.03

Case no., Case number; Pre, Preoperative; 1 M, 1 month postoperatively; 6 M, 6 months postoperatively; 18 M, 18 months postoperatively; PTA, Pure tone audiometry; DHI, Dizziness handicap inventory; BBS, Berg balance scale; THI, Tinnitus handicap inventory; HYN, Hypertension; TC, Total cholesterol; FBG, Fasting blood glucose; L, Left; R, Right; F, Female; M, Male; NT, No tinnitus; Y, Yes; N, No. Hypertension cut-off point was ≥140/90 mm Hg. Reference ranges for total cholesterol: normal was 3.10–5.70 mmol/L; reference ranges for fasting blood glucose: normal was 3.90–6.10 mg/dL.

The vertigo symptoms and imbalance resolved in all nine patients, 3 days and 3 weeks postoperatively, respectively. The successful control rate of vertigo was 100% (9/9) in the average 23-month postoperative follow-up evaluation, with complete control rate of 88.89% (8/9) and substantial control rate of 1.11% (1/9).

**Figure 1:**
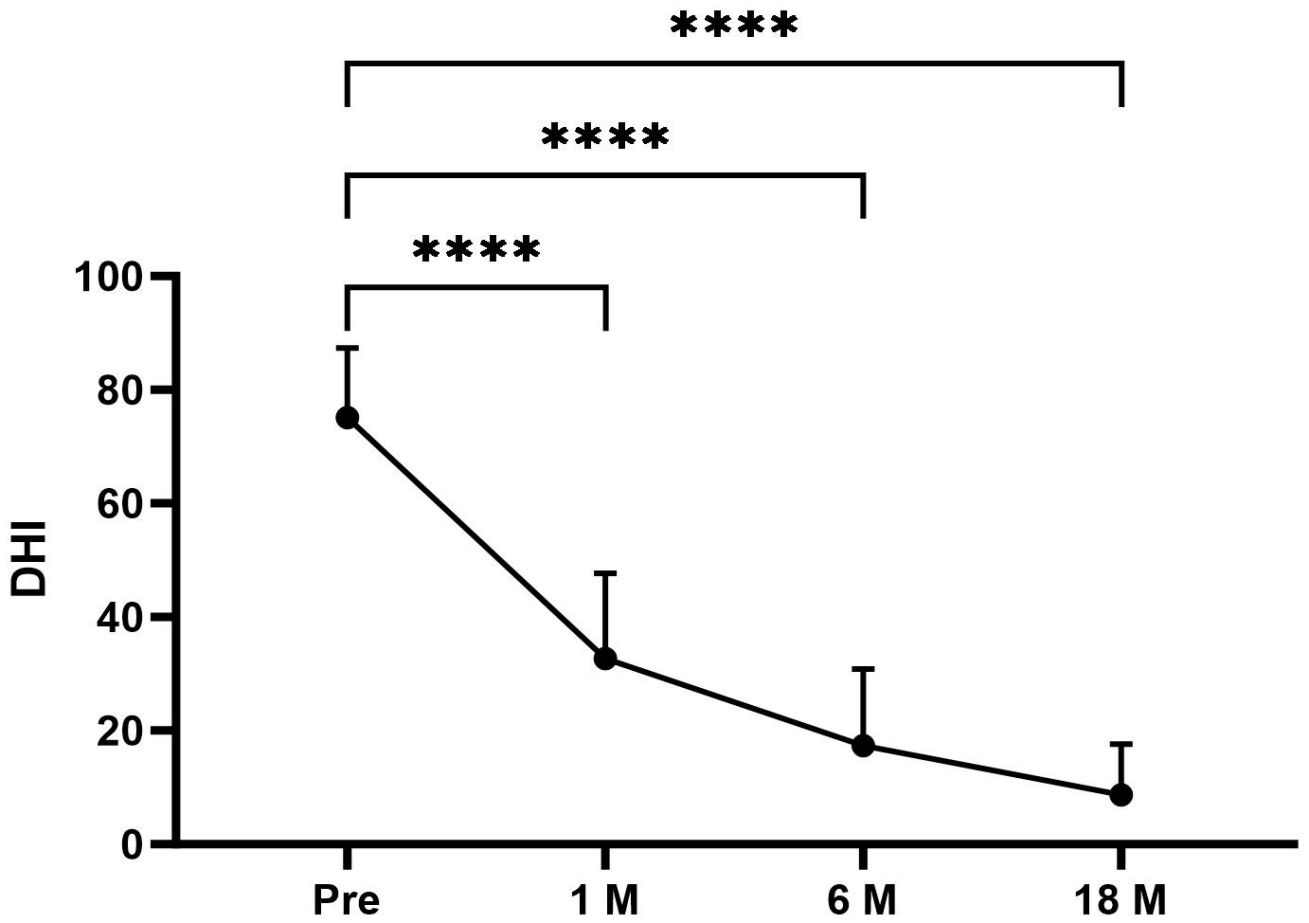
The average DHI scores at each period were as follows: preoperative period, 75.11 ± 12.252; 1 month postoperatively, 32.67 ± 15.033; 6 months postoperatively, 17.33 ± 13.528; and 18 months postoperatively, 8.67 ± 8.944. Compared to the average DHI scores for the preoperative period, the scores for the 1-, 6-, and 18-month postoperative periods after surgery were significantly lower. Pre, Preoperative; 1 M, 1 month postoperatively; 6 M, 6 months postoperatively; and 18 M, 18 months postoperatively. Statistical differences were determined using Student’s *t*-test. The ns denotes a statistically significant difference at a *p* > 0.05, ^∗^ denotes a statistically significant difference at a *p* < 0.05, ^∗∗^ denotes a statistically significant difference at a *p* < 0.01, ^∗∗∗^ denotes a statistically significant difference at *p* < 0.001, and ^∗∗∗∗^ denotes a statistically significant difference at a *p* < 0.0001.

**Figure 2:**
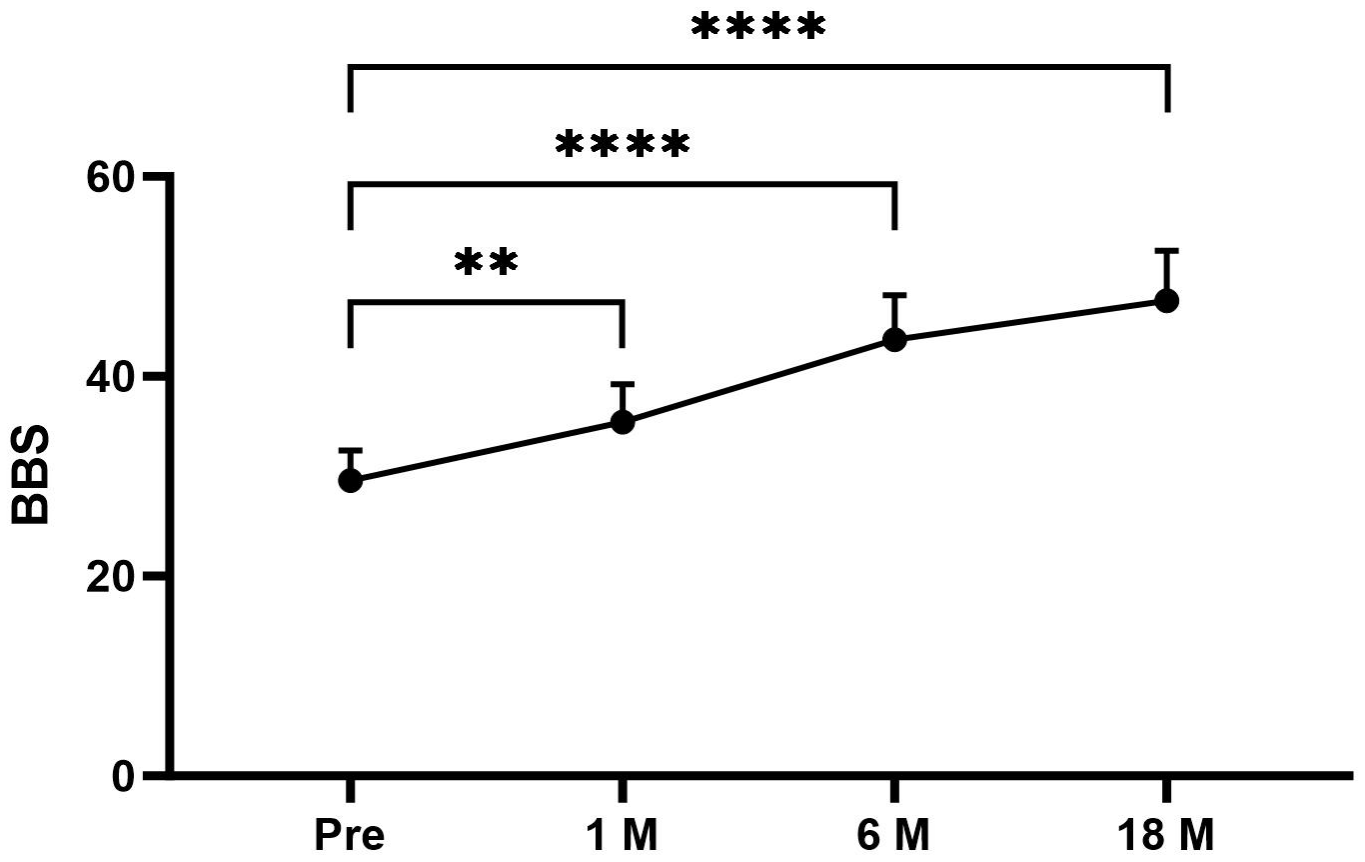
The average BBS scores at each period were as follows: preoperative period, 29.56 ± 3.005; 1 month postoperatively, 35.44 ± 3.779; 6 months postoperatively, 43.67 ± 4.444; and 18 months postoperatively, 47.56 ± 5.028. Compared to the average BBS scores for the preoperative period, the scores for the 1-, 6-, and 18-month postoperative periods after surgery were significantly higher. Pre, Preoperative; 1M, 1 month postoperatively; 6M, 6 months postoperatively; and 18M, 18 months postoperatively. Statistical differences were determined using Student’s *t*-test. The ns denotes a statistically significant difference at a *p* > 0.05, ^∗^ denotes a statistically significant difference at a *p* < 0.05, ^∗∗^ denotes a statistically significant difference at a *p* < 0.01, ^∗∗∗^ denotes a statistically significant difference at a *p* < 0.001; and ^∗∗∗∗^ denotes a statistically significant difference at a *p* < 0.0001.

The average DHI and BBS total scores pre- and postoperatively in different times periods were as follows (Figures 1, 2): pretreatment period (DHI, 75.11 ± 12.252; BBS, 29.56 ± 3.005); 1 month postoperatively (DHI, 32.67 ± 15.033; BBS, 35.44 ± 3.779); 6 months postoperatively (DHI, 17.33 ± 13.528; BBS, 43.67 ± 4.444); and 18 months postoperatively (DHI, 8.67 ± 8.944; BBS, 47.56 ± 5.028). In all periods post the triple semicircular canal occlusion combined with endolymphatic sac decompression, the DHI and BBS scores were significantly less post-TSCO combined with ESD compared to preoperatively in all time periods.

**Figure 3:**
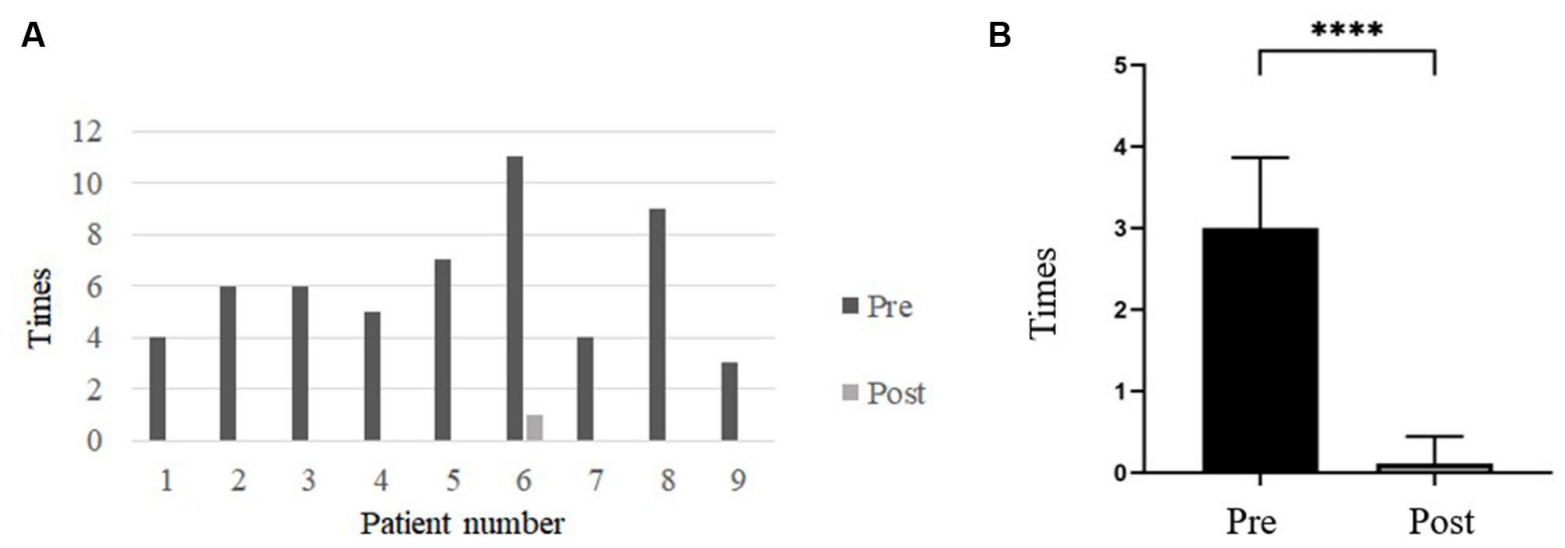
**(A)** Bar graph comparing the frequency of dizziness attacks before and after surgery. After surgery, the frequency of vertigo attacks was significantly reduced. **(B)** The frequency of vertigo attacks before and after TSCO with ESD. The frequency of vertigo attacks was 3.0 ± 0.866 times/month before surgery and 0.11 ± 0.333 times/month after surgery. The frequency decreased significantly after TSCO with ESD. Pre, Preoperative; Post, Postoperative. ^∗∗∗∗^Denotes a statistically significant difference at a *p* < 0.0001.

The frequency of vertigo attacks was 3.0 ± 0.866 times per month before and 0.11 ± 0.333times per month after undergoing TSCO with ESD. The frequency (%) of vertigo was compared pre- and post-operatively for a period of 18 months before and after treatment (Figure 3). The frequency of vertigo attacks decreased significantly after surgery. Eight of nine patients (88.89%) achieved complete vertigo control.

All patients underwent cervical cVEMP and ocular oVEMP testing. One of nine patients (11.11%) and two of nine patients (22.22%) had abnormal cVEMP results in the affected ear pre- and post-operatively, respectively. The oVEMP could not be elicited in three of nine patients (33.33%) before and after surgery. Preservation of VEMPs is beneficial to vestibular rehabilitation (15).

**Figure 4:**
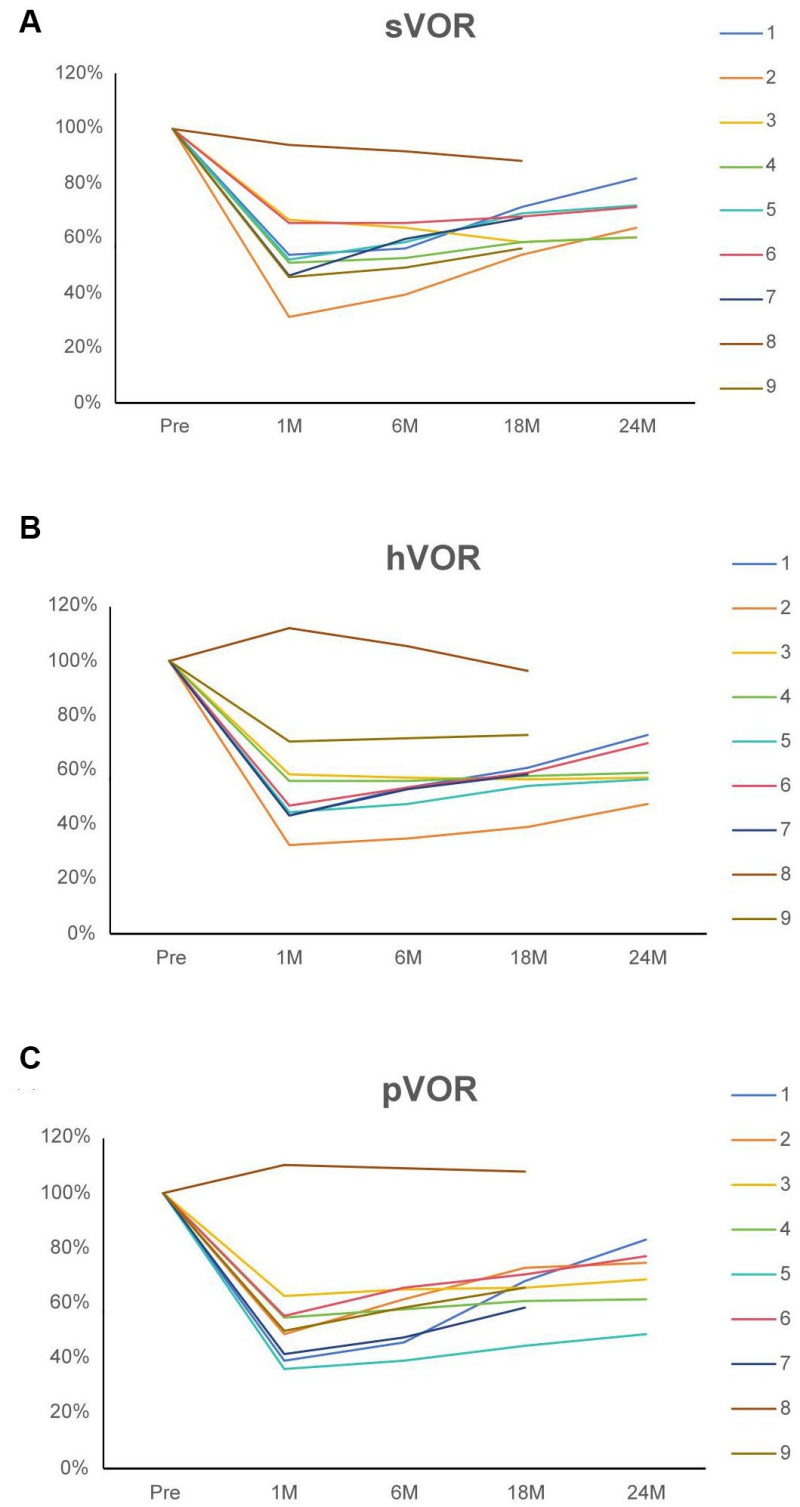
**(A)** The superior vestibulo-ocular reflex of the operated side for nine patients before and after surgical intervention. **(B)** The horizontal vestibulo-ocular reflex of the operated side for nine patients before and after surgical intervention. **(C)** The posterior vestibulo-ocular reflex of the operated side for nine patients before and after surgical intervention. The values are normalized to the preoperative value (100%). Each patient is identified by a color code and number. Patients 6–9 had follow-up evaluations for <2 years. sVOR, Superior vestibulo-ocular reflex; hVOR, Horizontal vestibulo-ocular reflex; pVOR, Posterior vestibulo-ocular reflex; Pre, Preoperative; 1 M, 1 month postoperatively; 6 M, 6 months postoperatively; 18 M, 18 months postoperatively; and 24 M, 24 months postoperatively.

The caloric test revealed that surgery induced canal paresis on the affected side in all patients. A decline in the v-HIT vestibulo-ocular reflex (VOR) gain of all plugged semicircular canals was demonstrated in the majority of patients. The vestibular results of the plugged semicircular canals are presented in Figure 4. There was an initial decline in the v-HIT VOR gain of all ipsilateral semicircular canals in eight patients, with the exception of patient 8. Considering the low preoperative v-HIT VOR gain measurement value, it is possible that artifacts affected the accuracy of the preoperative test results in patient 8. Ideally, the patient’s preoperative v-HIT VOR gain should have been higher than the measured value. Consequently, there appears to be an abnormal situation of relatively increased VOR after surgery. Recovery varied among patients during follow-up.

**Figure 5:**
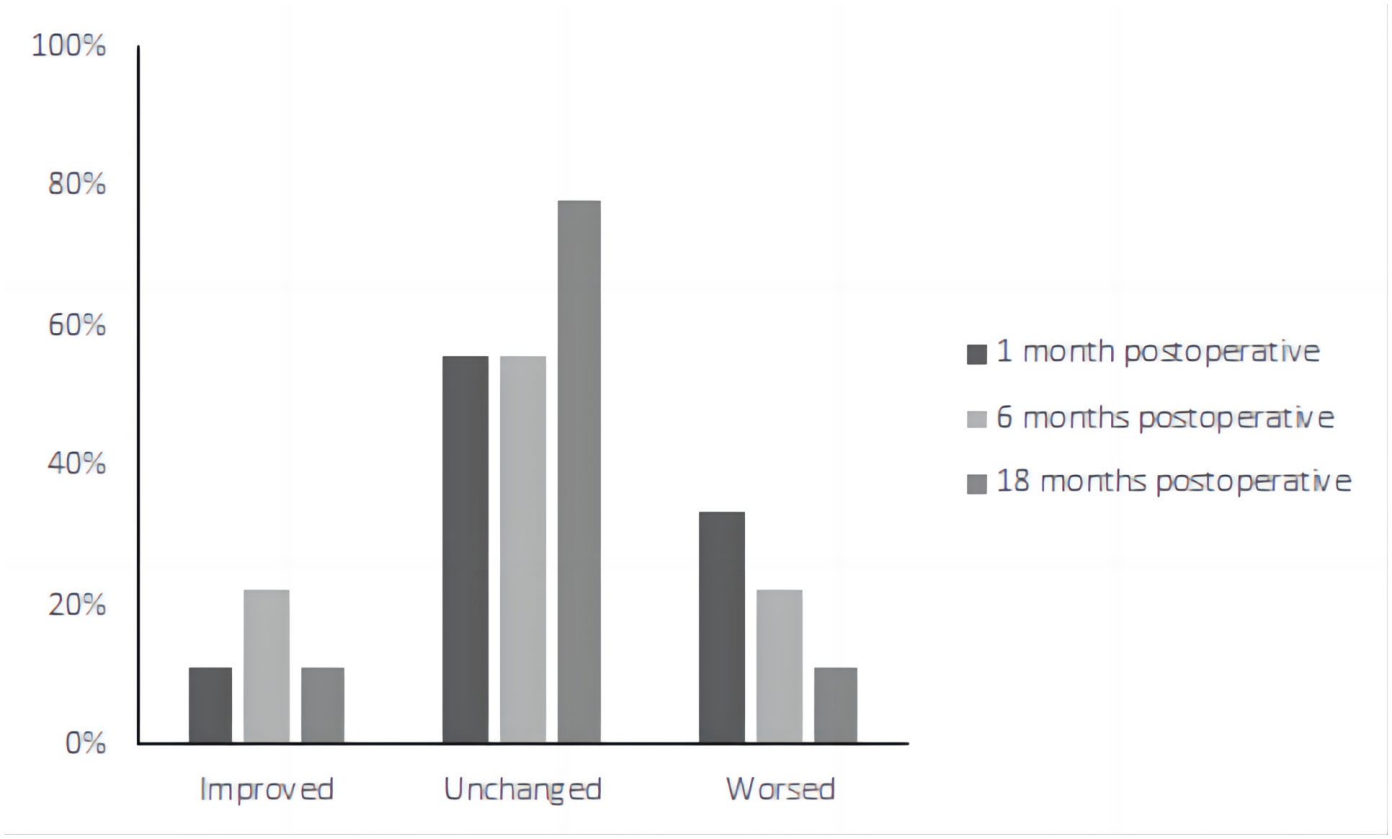
The pure tone audiometry results of nine patients postoperatively. The rate of hearing loss was 33.33% (3/9) in the first month after surgery. At the 6-month follow-up evaluation, the PTA was decreased >10 dB compared to preoperative testing in two patients (patients 5 and 8).

Preservation of hearing after TSCO with ESD surgery is a significant concern for patients. The rate of hearing loss was 33.33% (3/9) in the first month after surgery (Figure 5). At the 6-month follow-up evaluation, the PTA was decreased >10 dB compared to preoperative testing in two patients (patients 5 and 8). This improvement in hearing could possibly be attributed to a decrease in the pressure of the inner ear membrane labyrinth after surgery.

**Figure 6:**
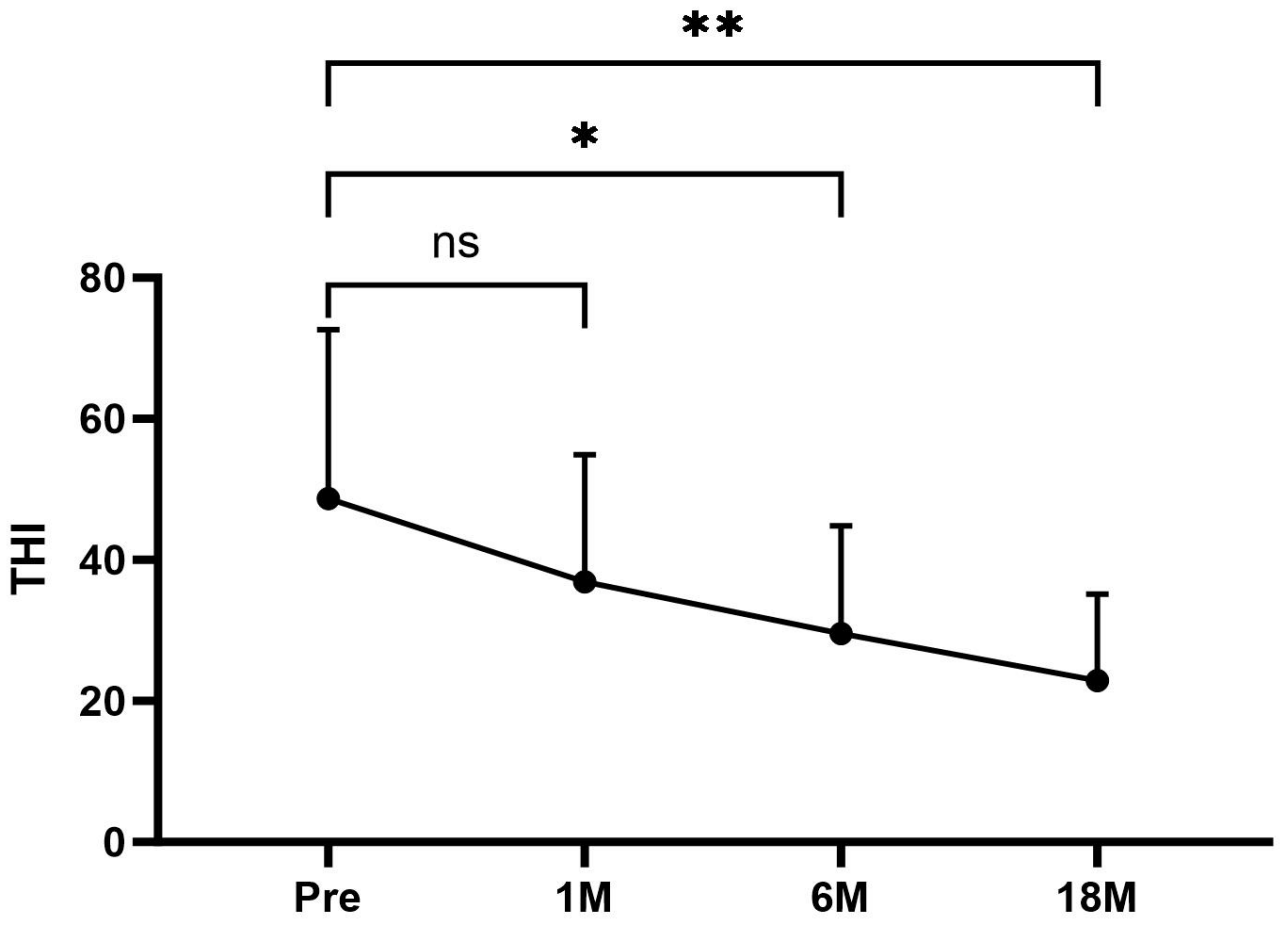
The average THI scores at each period were as follows: preoperative period, 48.67 ± 23.979; 1 month postoperatively, 36.89 ± 18.003; 6 months postoperatively, 29.56 ± 15.257; and 18 months postoperatively, 22.89 ± 12.293. Compared to the average THI scores for the preoperative period, the scores for the 6 and 18 months postoperative periods after surgery were significantly lower. There was no significant difference in the scores 1 month postoperatively compared with the average THI scores before surgery. Pre, Preoperative; 1 M, 1 month postoperatively; 6 M, 6 months postoperatively; and 18 M, 18 months postoperatively.

Of the eight patients who had tinnitus symptoms before surgery, all eight had significant relief after undergoing the surgical procedure (Figure 6). The average THI before and after surgery in different time periods was as follows (Figure 6): pre-treatment period (THI, 48.67 ± 23.979); 1 month postoperatively (THI, 36.89 ± 18.003); 6 months postoperatively (THI, 29.56 ± 15.257); and 18 months postoperatively (THI, 22.89 ± 12.293).

## Discussion

The characteristic features of Meniere’s disease include fluctuating sensorineural hearing loss accompanied, tinnitus, and episodic vertigo. The hallmark feature of this disease is the fluctuation of symptoms, with periods of waxing and waning. The episodes of vertigo can last from several minutes-to-hours, and there may be positional vertigo in between and during attacks. Additionally, many patients experience a sense of pressure or fullness in the ear, along with different forms of tinnitus (3).

Meniere’s disease is thought to be caused by an excess of endolymph in the inner ear resulting from an imbalance in endolymph production and resorption of endolymph in the endolymphatic sac (16). Moreover, saccular hydrops is associated with sensorineural hearing loss (17).

The vertigo experienced in association with MD is attributed to a sudden surge in endolymphatic pressure, leading to a reversal of endolymphatic fluid flow toward the utricle and semicircular canal via Bast’s valve (18).

The clinical course of Meniere’s disease is unpredictable, which is attributed to the capricious nature of the disorder. Approximately 70% of patients respond positively to conservative medical management, while the remaining 30% respond poorly and often necessitate surgical therapy to control their symptoms (19).

In 1927, Portmann introduced the concept of endolymphatic sac surgery. The procedure involves a mastoidectomy to incise the endolymphatic sac, facilitating the drainage of endolymph (20). ESD is considered effective and safe for patients with intractable MD by decompressing the inner ear in cases of endolymphatic hydrops. ESD causes a reduction in endolymphatic pressure, which is still controversial as the rationale for performing ESD (21). Chung et al. (22) examined 15 postmortem temporal bone specimens from patients who underwent endolymphatic sac mastoid drainage and discovered that endolymphatic hydrops persisted in all patients, which raises doubts about the effectiveness of endolymphatic sac surgery. Despite the controversy, endolymphatic sac surgery is still recommended by the Clinical Practice Guidelines published by the American Academy of Otolaryngology–Head and Neck Surgery Foundation for patients who have failed conservative treatment (23). In addition to reducing pressure of endolymphatic, endolymphatic sac surgery can also affect endolymphatic sac secretory function. Gibson proposed that abnormal accumulation of fluid in the inner ear triggers the endolymphatic sac reaction, which will secrete osmotically active glycoproteins so that the endolymph fluid will flow quickly from the inner ear to the sac, thus causing vertigo attacks (24).

Destruction of sac function potentially improves vertigo control. This finding may be due to the elimination of glycoprotein secretion into the sac, which facilitates regulation of rapid fluid changes and ultimately decreases vertigo symptoms (25). Sood et al. (26) searched PubMed databases to retrieve articles on sac decompression. The findings suggest that endolymphatic sac surgery is effective in controlling vertigo in 79.3% of patients with intractable MD. Brinson et al. (27) reported that the rate of vertigo control was 66% in 54 patients who received ESD with an average follow-up period of 31 months. Li et al. (28) evaluated 56 patients with Meniere’s disease who underwent ESD with a follow-up duration of 18–24 months. Li et al. (28) showed that ESD was effective in reducing the incidence and severity of vertigo attacks with significant improvement in 80.36% of patients.

Semicircular canal occlusion is an effective surgical treatment for BPPV. The purpose of this treatment is to ablate movement of endolymph in the canals. Yin et al. (6) recently applied TSCO as an alternative treatment in patients with intractable MD. In their pilot review, Yin et al. (6) assessed three patients who had previously undergone unsuccessful endolymphatic sac surgery and subsequently underwent TSCO. At the 2–5 years follow-up evaluations, two patients reported complete remission of vertigo symptoms and one patient had significant improvement. This finding indicated that TSCO effectively relieved vertigo symptoms in patients with intractable MD. TSCO is currently used with good curative effect. Based on the literature, the reported vertigo control rate ranges from 96.7–100% (6, 29). The possible mechanism underlying might be associated with a reduction in movement of endolymph and minimizing hair cell stimulation during angular movement (7).

Despite the high rate of TSCO effectiveness in the control of vertiginous symptoms, some patients experience hearing loss after surgery. Zhang et al. (8) reported that the rate of hearing loss was 26.3% after TSCO during 2 years of follow-up, which indicated that plugging semicircular canals was a risk factor for hearing loss. It is not clear why hearing deteriorates over time after semicircular canal occlusion. There are some factors, including lymph leaks and infections, that are considered to potentially induce hearing loss (30). Moreover, endolymphatic sac decompression surgery is a functional procedure that enables a high rate of hearing preservation. Therefore, the combination of TSCO and ESD is an ideal approach because each procedure can complement the drawbacks of the other.

Endolymphatic sac decompression can minimize potential inner ear damage, including aggravation of endolymphatic effusions, due to increased pressure in the long-term resulting from plugging of semicircular canals (31). TSCO can block signal conduction in the vertigo conduction pathway and enhance vertigo control. It is appropriate to combine TSCO and ESD to complement the drawbacks of each procedure alone. Therefore, it is possible to achieve a higher success rate in controlling vertigo, while minimizing the risk of hearing loss and allowing a balance to be achieved between vertigo control and hearing preservation.

Regarding the phenomenon in which all patients display spontaneous nystagmus immediately after surgery and some patients complain of minor imbalance for several weeks, Zhang et al. attributed this to the “quiescent stage” of the end organs. Zhang et al. explained that the resting discharge of the end organs is temporarily affected, but eventually recovers as the end organs remain morphologically undamaged (8). The caloric test demonstrated that surgery induced canal paresis on the affected side in all patients, indicating that TSCO with ESD effectively controlled vertigo by eliminating semicircular canal function (8).

The v-HIT test demonstrated a decline in semicircular canal function, resulting in a higher threshold for vertigo attacks (32).

Vestibular rehabilitation protocols accelerate the process of vestibular compensation after TSCO with ESD, thereby facilitating recovery of postoperative imbalance.

The precise mechanism explaining the recovery of VOR remains unclear. This could be due to incomplete canal occlusion, which does not appear to impact treatment success, or it could be linked to the achievement of vestibular compensation (16). ITG was utilized to eliminate vestibular function in the affected ear and decrease the frequency and intensity of vertigo episodes; however, there may be a lingering imbalance afterwards. It is worth noting that vestibular hair cells are more susceptible to gentamicin compared to cochlear hair cells. This discrepancy in toxicity allows for the preservation of hearing in certain situations while simultaneously alleviating vertigo (33).

Intratympanic steroid has been shown to effectively control vertigo attacks while minimizing the risk of hearing loss. Steroids possess anti-inflammatory properties and when bound to specific receptors regulated transmitter levels and inhibited exudation. Additionally, ITS impacted transmembrane transport and the transport of water-salt electrolytes, thereby maintaining endolymph balance (34).

In the treatment of MD, ITG was more effective than ITS in reducing the number of vertigo attacks (35). Compared to intratympanic injection of gentamicin, TSCO with ESD had beneficial effects in the treatment of MD. Patel et al. (36) reported that the average control rate of vertigo is 83.0% after ITG. Although less invasive than surgery, ITG impaired hearing and caused severe long-term vestibular sequelae (37).

Triple semicircular canal occlusion and vestibular neurotomy both have a high control rate of vertigo. Compared to vestibular neurotomy, TSCO has several advantages, such as less trauma, faster recovery, and lower risk of facial nerve injury. While vestibular neurectomy has proven to be effective in controlling vertigo, it is important to consider the potential risks and complications associated with the procedure, some of which can be life-threatening (38). Additionally, the process of vestibular compensation following vestibular neurectomy is more intricate and can result in a longer delay before patients can return to work (39). Due to the rigorous selection process for TSCO and ESD surgery, only a limited number of patients were enrolled in the current study. Nevertheless, the current evaluation provides valuable insight into the treatment of frequent vertigo in patients with MD. Moreover, the indications for surgery in patients with MD were based on current hearing staging. We are of the opinion that the indications for surgery should focus more on the degree of vertigo. If Tumarkin’s otolithic crises occur, surgery is recommended.

## Conclusion

Triple semicircular canal occlusion with ESD was a safe technique that effectively managed frequent vertigo in patients with MD, especially when other measures have failed.

## Data availability statement

The original contributions presented in the study are included in the article/supplementary material; further inquiries can be directed to the corresponding author.

## Ethics statement

The studies involving humans were approved by the Third Affiliated Hospital of Sun Yat-sen University. The studies were conducted in accordance with the local legislation and institutional requirements. Written informed consent to participate in this study was provided by the patients/participants. Written informed consent was obtained from the individual(s) for the publication of any potentially identifiable images or data included in this article.

## Author contributions

JT: Data curation, Writing – original draft, Writing – review & editing. GY: Writing – original draft. QZ: Data curation, Writing – review & editing. SZ: Project administration, Writing – review & editing. XZ: Project administration, Writing – review & editing. YL: Supervision, Writing – review & editing.
